# Biomarkers for Lung Nodule Risk Stratification: Are We There Yet?

**DOI:** 10.3390/diagnostics16121861

**Published:** 2026-06-16

**Authors:** Sushan Gupta, Swati Jain, Krupa Shingada, Aikta Rajput, Jonathan S. Kurman

**Affiliations:** 1 Department of Pulmonary & Critical Care, Medical College of Wisconsin, Milwaukee, WI 53226, USA; sgupta@mcw.edu (S.G.); swjain@mcw.edu (S.J.); kshingada@mcw.edu (K.S.); 2 Northwell Health, New York, NY 11042, USA; arajput@northwell.edu

**Keywords:** lung nodules, pulmonary nodules, biomarker, risk stratification

## Abstract

The number of pulmonary nodules detected is steadily increasing. While some are low or high risk, most are indeterminate. Within the indeterminate group, the vast majority are benign. Statification of indeterminate pulmonary nodules (IPNs) is challenging. Validated risk calculators are not routinely employed in clinical practice because they are cumbersome. Most clinicians rely upon clinical gestalt. This strategy often results in unnecessary biopsies, which are costly, stressful, and have the potential for iatrogenic injury. There are a number of commercially available lung cancer risk biomarkers, which each have their own strengths and weaknesses. Barriers to widespread adoption include lack of guideline support, clinical practice inertia, and limited clinical utility data. Upcoming multicenter trials aim to provide high quality data that could lead to guideline incorporation.

## 1. Introduction

Lung cancer is the leading cause of cancer-related deaths. Survival decreases dramatically at advanced stages, underscoring the critical importance of early detection and diagnosis. Approximately 1.6 million patients are diagnosed with incidentally detected pulmonary nodules every year on chest computed tomography (CT) scan images in the United States (US) [1]. Most of these nodules (≥95%), whether incidentally or screening-detected, are benign [2]. Despite this, many patients undergo unnecessary invasive procedures to rule out cancer because our current stratification approaches are suboptimal, especially for intermediate pulmonary nodules (IPN) [3]. Approximately 4000 IPNs are identified in the US daily. There is significant heterogeneity in guideline recommendations and in the implementation of IPN management. More than 40% of the overall cost of lung cancer management can be attributed to benign nodules managed with an invasive procedure [4]. An ideal evaluation of an individual with a pulmonary nodule would expedite therapy for malignant nodules and minimize testing in those with benign nodules [1]. It is neither feasible nor recommended to biopsy all nodules; a balanced approach is critical. Currently, we are doing a poor job of that and erring on the side of caution. This has a direct economic impact and constrains limited resources.

### Review of Current Guidelines (ACCP, Fleischner, Lung Rads, etc.)

Several societies have developed guidelines to assist with the evaluation of an individual with a pulmonary nodule. These guidelines vary in their scope and focus, and some are specific to certain geographic regions and health systems [1]. Guidelines differ in their use of different thresholds to estimate the probability of malignancy to provide management recommendations. In general, smaller pulmonary nodules are more likely to be benign. Pulmonary nodules can be categorized as small solid (<8 mm), larger solid (≥8 mm), and subsolid. Subsolid nodules are divided into ground-glass nodules (no solid component) and part solid (both ground-glass and solid components). As a group, nodules < 6 mm have approximately a 1% chance of malignancy. This increases slightly to 1–2% for nodules that are 6–8 mm. Nodules that are 6–8 mm can be followed with a repeat CT chest in 6–12 months, depending on clinical risk factors, imaging characteristics, and physician-assessed risk of malignancy. The treatment of individuals with a solid pulmonary nodule ≥ 8 mm is based on the estimated probability of malignancy, comorbidities, and patient preference. Management options include surveillance imaging (monitoring for nodule growth with CT or positron emission tomography (PET) scans), non-surgical biopsy with bronchoscopy or transthoracic needle biopsy, or surgical resection. Part-solid nodules are managed according to the size of the solid component. Larger solid nodules have a higher risk of malignancy. Current bronchoscopy and transthoracic needle biopsy methods have a sensitivity of 70–90% for diagnosing lung cancer [1].

Management of pulmonary nodules is guided by classifying them into three categories according to the risk of malignancy. Those with low risk (<5%) can be monitored with imaging, while those with high risk (>65%) should proceed directly to surgery [1]. Intermediate risk (5–65%) nodules often undergo additional diagnostic testing. Nodules in this category have the greatest need for more standardized management, given the heterogeneity in guideline recommendations and their implementation. Factors that influence clinical decisions include the anticipated diagnostic yield of a non-surgical biopsy, patient comorbidities that impact the safety of the procedure, likelihood of benefit from establishing a diagnosis, and patient preference [1].

Each set of guidelines utilizes a different threshold to estimate the probability of malignancy to provide management recommendations. This is further complicated by variable compliance among clinicians with guideline recommendations and by patients’ willingness to adhere to them [1]. In one study of 197 patients with pulmonary nodules, 88 (44.7%) received care inconsistent with guidelines, e.g., next surveillance imaging before or after the interval recommendation in the guidelines, or a non-surgical biopsy instead of surveillance imaging [5]. Features associated with non-compliance included inappropriate radiologist guidance, overestimation of relative risk, nodule detection during inpatient stays or perioperative visits, and other factors [1]. Expected benefits of guideline adherence include cost savings by avoiding unnecessary invasive procedures and their associated iatrogenic complications, as well as expediting the diagnosis and initiation of treatment for malignant nodules.

Currently, there is significant heterogeneity in management recommendations per different guidelines, depending on how the nodule was identified. Some may be outdated at this point. Despite advances in biomarker identification, most current guidelines do not incorporate these noninvasive tests into initial risk stratification and decision-making. There is a need for improved risk stratification to ensure that the appropriate nodule is biopsied at the appropriate time.

## 2. Biomarkers

Novel risk stratification tools have been developed to meet this need. Multiple biomarkers have been developed in recent years and may prove valuable in addressing this gap and supporting management decisions in this area. The National Institutes of Health (NIH) defines a biomarker as a characteristic that can be objectively measured and used to evaluate normal biological processes, pathogenic processes, or pharmacological responses to a therapeutic intervention. Most biomarkers rely upon biologic samples from patients in the form of blood or sputum. Others rely solely upon radiographic characteristics. This manuscript reviews various biomarkers (as risk-stratification tools) that are commercially available in the United States or under development (Table 1, Table 2, Table 3 and Table 4). Biomarkers in development and a detailed analysis of the mechanisms of action of various biomarkers are beyond the scope of this manuscript but have been previously discussed by McGann et al. [6].

Current biomarkers rely upon serum proteins, quantitative image analysis (radiomics), exhaled volatile organic compounds, and bronchial or nasal epithelium, among others [3]. Commercially available biomarkers include Nodify XL2, Nodify CDT, LungLifeAI, CyPath Lung, and the Lung Cancer Predictor (LCP) (Table 1). All the above-mentioned biomarkers have undergone clinical accuracy studies. Nodify XL2 and CyPath Lung have reported negative predictive values (NPV) of 98% and 99%, respectively.

Biomarkers have the potential to reclassify IPNs as low- or high-probability, thereby reducing unnecessary and costly procedures and shortening the time to treatment of malignant nodules. In 2017, the American Thoracic Society (ATS) issued guidance on the evaluation of lung cancer biomarkers [7]. It emphasized differentiating between biomarker performance and clinical utility. The clinical utility of a novel biomarker requires demonstration of clinical benefit in clinical care. It can either reduce invasive procedures in patients with benign disease or decrease time to diagnosis and treatment in patients with malignancy.

Although many biomarkers have been developed, only a few have been commercialized, and even fewer are routinely used in clinical practice. The barriers to routine use include cost, accessibility, clinical practice inertia, and limited evidence of clinical utility, including studies showing improved patient outcomes. Clinical utility of a novel biomarker requires demonstration of clinical benefit when it is used in clinical care. There is a need for rigorously conducted clinical research studies to fully evaluate the clinical effectiveness of biomarkers and AI tools for risk stratification and to clearly define their role in lung nodule management algorithms. Until then, guideline incorporation will not be assured.

## 3. Commercially Available Biomarkers

1. Nodify XL2 (Biodesix) (formerly Integrated Dx Expresys Lung) is an integrated classifier test that was introduced commercially in its current form in 2019. It combines a blood-based assay with clinical and radiologic criteria to identify nodules that are likely benign. The assay employs multiple reaction monitoring mass spectrometry (MRM-MS) to measure the ratio of two proteins, LG3BP and C163A. The turnaround time for the test result is 3–5 days. Results are reported as a percentage. The test is designed for utilization before a nodule is biopsied to help rule out malignancy.
  Eligibility criteria include age ≥ 40 years, nodules 8 to 30 mm in size, <50% risk of malignancy per the Mayo Clinic solitary pulmonary nodule malignancy risk score, no history of lung cancer ever, and no history of non-lung cancer in the last 5 years.
  The test was clinically validated in the PANTOPIC (Pulmonary Nodule Plasma Proteomic Classifier) trial, which demonstrated a sensitivity of 97%, a specificity of 44%, and a negative predictive value (NPV) of 98% for distinguishing benign from malignant nodules [8].

2. Nodify CDT (Biodesix) (formerly Oncimmune EarlyCDT) is a blood-based assay that was introduced in its current form in 2020. It utilizes enzyme-linked immunosorbent assay (ELISA) to identify seven autoantibodies associated with tumor antigens (p53, NY-ESO-1, MAGE A4, GBU4-5, CAGE, HuD, and SOX2). The test result is available within one day and is reported as a percentage. The test is designed to be used prior to consideration of biopsy and can help to establish malignancy as a possibility.
  Eligibility criteria for this test include age ≥ 40 years, nodules 8 to 30 mm in size, less than 65% risk of malignancy per the Mayo Clinic solitary pulmonary nodule malignancy risk score, and no history of cancer.
  This test was assessed for clinical accuracy in a study by Massion et al. [9]. When test results were combined with other available risk models, specificity was >90% and positive predictive value was >70%.

3. LungLife AI (Circulogene) (formerly LungLB by LungLife AI) is a blood-based assay that was introduced commercially in 2024. It utilizes immunomagnetic depletion combined with a 4-color fluorescence in situ hybridization (FISH) to detect circulating genetically abnormal cells (CGACs). CGACs are further classified as advanced based on specific anomalies detected via FISH. The test results are available in approximately 5–7 days and the results are binary (either increased or decreased risk). The test is designed for use prior to consideration of biopsy and can help to rule in malignancy.
  Eligibility criteria for this test include age < 80 years, upper lobe location, and nodule size < 15 mm.
  This test was assessed for clinical accuracy in a study by Tahvilian et al. and demonstrated a sensitivity of 77% and a specificity of 72% for patients with advanced CGACs [10].

4. CyPath Lung (BioAffinity) is a sputum-based assay that was first introduced commercially in 2023. It combines automated flow cytometry (FCM) data analysis of induced sputum samples with machine learning techniques to detect tumor-associated antigens. Age is incorporated into the algorithm. Results are typically available in three days and reported as a percentage. The test is designed to be utilized prior to consideration of biopsy and can help to rule out malignancy.
  Eligibility criteria for this test include age > 50 years, nodules 6 to 20 mm in size, and current or former smoking.
  This test was assessed for clinical accuracy in a study by Lemieux et al. demonstrating a sensitivity of 92%, specificity of 87% and an NPV of 99% for all nodules < 20 mm [11].

5. The Lung Cancer Prediction Convolutional Neural Network (LCP-CNN) (Optellum) is an artificial intelligence-based computer-aided diagnosis (CAD) model first introduced commercially in 2021. It utilizes radiomics to analyze CT chest data to provide malignancy risk estimates (from 0 to 100). The Lung Cancer Prediction (LCP) Score is derived from these risk estimates. It categorizes malignancy risk on a decile scale (range 1 to 10), with a score of 10 indicating nodules of the highest risk. The results are available almost instantly.
  Eligibility criteria include age > 35 years, nodules 5 to 30 mm, and no history of lung cancer in the past five years.
  A study by Kim et al. demonstrated that use of CAD significantly improved sensitivity, specificity, and interobserver agreement at malignancy risk thresholds of 5% and 65% [12].

In addition to the biomarkers described above, several have been discontinued for various reasons including economic viability (Table 5). Percepta Genomic Sequence Classifier (Veracyte) was commercially introduced in 2021 but was withdrawn from the market in 2024. It combined whole-transcriptome RNA sequencing of a bronchial epithelium brush sample obtained during bronchoscopy with clinical factors. The Reveal Lung Nodule Characterization Score and Knodule ID (MagArray) were both derived using an algorithm that incorporates clinical factors and blood-based assays to detect proteins associated with lung cancer. These were discontinued in approximately 2023.

## 4. Discussion

Lung cancer remains the leading cause of cancer-related deaths worldwide. For low- and high-risk pulmonary nodules, guidelines are relatively consistent in their recommendations. IPN management remains challenging due to heterogeneity in recommendations and the need for at least some degree of individualized management.

### 4.1. Need for Biomarkers

Lack of a highly sensitive non-invasive screening test with wide applicability may contribute to heterogeneity in guideline recommendations for IPNs. Although low-dose CT (LDCT) imaging is beneficial in high-risk populations, defined as those ages 50–80 years who are current or former smokers, it excludes younger patients and those without a smoking history [13].

Moreover, the specificity of LDCT is approximately 73%, which likely reflects the frequent non-malignant etiologies of IPNs [14]. Unfortunately, many of these undergo unnecessary invasive biopsies, which contribute to high healthcare expenditure related to lung cancer. Medicare analyses estimate that benign nodules account for nearly 40% of the overall cost of lung cancer care, including invasive procedures [4].

This situation has created a desperate need for easily sampled, inexpensive biomarkers that can reliably identify patients with early lung malignancy, thereby mitigating cost and providing clinical benefit.

### 4.2. Key Similarities and Differences

Many of the biomarkers that have been developed incorporate a biologic specimen and clinical factors, whereas others rely solely on the biologic specimen. The products were validated in slightly different nodule populations, so not every test is applicable to every nodule. Like risk calculators based on clinical factors (Mayo model, Brock model, etc.), the correct biomarker must be utilized in the correct patient. The amount of data and the scientific rigor with which studies were performed differ dramatically among the commercial biomarkers. Importantly, none of the biomarkers discussed below have been compared head-to-head. Any comparator statements should be assumed to be referring to the standard of care, which is typically determined at the individual provider level.

Nodify XL2 (Biodesix) uses a blood-based assessment of two proteins, LG3BP and C163A, combined with five clinical parameters to reclassify pulmonary nodules between 8 and 30 mm. In the PANOPTIC study, the relative abundance of LG3BP and C163A proteins in an integrated model distinguished benign from malignant pulmonary nodules with more than 95% sensitivity and negative predictive value [8]. The ORACLE study assessed clinical utility in a multicenter prospective nonrandomized manner [15]. Use of Nodify XL2 avoided one invasive procedure per seven patients tested, resulting in a 74% relative risk reduction [15]. These findings were replicated by Kheir et al. in a multicenter retrospective analysis [16]. More rigorous assessment of clinical utility is being conducted in the ongoing ALTITUDE trial.

Although no longer available, Reveal and Knodule ID (MagArray) have applied machine-learning techniques to an array of seven plasma proteins (carcinoembryonic antigen, epidermal growth factor receptor, neutrophil-activating protein 2, pro-surfactant protein B, C-X-C motif chemokine ligand 10, the receptor for advanced glycation end products, and tissue inhibitor of metalloproteinases-1) together with seven clinical variables, to develop a risk reclassification model. This model stratified IPNs into high- and low-risk categories, improving the true-positive-to-false-negative ratio [17]. A similar AI-based approach is used by Optellum, which employs the Lung Cancer Prediction Convolutional Neural Network (LCP-CNN). This AI-based computer tool provides a more accurate estimate of malignancy risk based on CT scan findings. It increased the average diagnostic discrimination for malignant IPNs, raising the AUC from 0.85 (without the AI tool) to 0.92 (*p* < 0.001). Among patients with IPNs, computer-assisted diagnosis also improved reader average AUC, sensitivity, and specificity for malignancy identification [18].

An alternative strategy involves identifying tumor-associated antibodies, which may offer higher specificity and the potential for earlier detection through immune signal amplification. Nodify CDT (Biodesix) uses an indirect ELISA to evaluate a panel of seven autoantibodies against seven lung cancer-associated antigens. In a recent validation study of 447 patients, Long et al. reported that the autoantibody panel achieved a specificity of 90% (95% CI, 85–93%), with even higher specificity in PET-positive patients. The test demonstrated substantially lower sensitivity (approximately 16%) than Nodify XL2 and a false-positive rate of 10% [19].

Another method for reclassifying IPNs involves identifying circulating transcriptomic biomarkers, especially microRNAs (miRNAs). These 20–22 nucleotide sequences, located in cancer-associated genes, are increasingly being studied as potential biomarkers for early lung cancer detection, including stage I and II NSCLC. Fehlmann et al. demonstrated that a panel of 14–15 miRNAs could differentiate lung cancer with excellent accuracy, with an AUC of 0.965 [20].

Detection of circulating tumor cells (CTCs), circulating tumor DNA (ctDNA), and circulating methylated DNA in blood has mainly been used to identify targetable mutations in thoracic oncology. LungLifeAI (Circulogene), however, is a blood-based assay that employs similar methodology to detect circulating genetically abnormal cells (CGACs) [10]. Data show that the test has relatively high specificity (96–100%) but more modest sensitivity (26–80%) [21,22,23]. The lower sensitivity may be partially mitigated by complementary ctDNA-based tests that detect fragmented tumor DNA in blood [24,25].

Newer machine learning models have incorporated more diverse approaches utilizing ctDNA, clinical data, and protein markers to stratify IPNs. ctDNA detection is challenging, however, as only about 30% of early-stage NSCLC cases release ctDNA in detectable amounts [26,27]. Identifying CTCs can also be challenging because of the mesenchymal transformation of cancer cells [28]. LungLifeAI utilizes immunomagnetic depletion combined with FISH to detect chromosomal instabilities in the form of genomic copy number variations (CNVs) in circulating genetically abnormal cells (CGACs) enriched from the peripheral blood. In a cohort of 151 patients, it achieved an AUC of 0.74 (95% CI, 0.66–0.84; *p* < 0.001), with 67.9% sensitivity and 74.4% specificity, compared with an AUC of 0.52 for the Mayo Clinic Model. Performance was even better in nodules < 2 cm (AUC 0.83), subsolid or nonsolid nodules (AUC 0.90), and stage I disease (AUC 0.80), with comparable performance regardless of histology [10]. Its greatest strength lies in its superior sensitivity in identifying stage I lung cancer, which has the greatest need for additional stratification.

CyPath Lung (BioAffinity) is a sputum-based assay that utilizes automated flow-cytometry to assess for specific antibodies and fluorescent tetra (4-carboxyphenyl) porphyrin (TCPP) that preferentially binds to cancer cells along with machine learning techniques. Lemieux et al. found that while the test fared well with all nodules (80% sensitivity and specificity), it excelled with those < 20 mm in size, where it was 92% sensitive and 87% specific (AUC of 0.94; 95% CI 0.89–0.99) [11].

The Percepta Genomic Sequencing Classifier (GSC) differed from the above tests as the epithelial samples were collected from the main stem bronchus using cytology brushing during bronchoscopy. This test was particularly helpful when bronchoscopy was inconclusive. It used genomic features from whole transcriptome RNA sequencing leveraging 23 genes and clinical factors [29]. It was first clinically validated in the Airway Epithelium Gene Expression in the Diagnosis of Lung Cancer cohorts (AEGIS I and II) trial [30]. It is the only product designed for use as both a “rule-in” and a “rule-out” test. In the validation study by Mazzone et al., the method demonstrated excellent negative predictive value: 29% of IPNs were down-classified to low risk, with 91% negative predictive value (NPV), and 54% of low-risk lesions were down-classified to very low risk, with >99% NPV. It also classified 12% of intermediate-risk lesions as high risk, with a positive predictive value (PPV) of 65.4% [31].

### 4.3. Role of Biomarkers in the Comprehensive Pulmonary Nodule Program

Biomarkers are only one piece of the puzzle whose diagnostic yield can be increased by combining single or multiple such biomarkers with clinical and imaging data to allow for more robust and reliable differentiation of pulmonary nodules. Each biomarker has shortcomings and limited diagnostic strength, limiting the isolated use of a single biomarker as a diagnostic tool. For example, the identification of CGACs relies on surface markers, which limits its ability to detect lung cancers that do not express these markers. This highlights that biomarkers are just part of the equation that can be completed with more robust machine-learning models that can leverage multiple data points, including clinical, imaging, and biomarker data, to allow high confidence in the risk stratification of patients with pulmonary nodules.

### 4.4. Barriers to Utilization

There are many barriers to the clinical utilization of biomarkers. The goal of biomarkers is to reduce the time to diagnosis without increasing the number of procedures being performed on benign nodules [3,7]. Unfortunately, despite extensive research in this field, these data are still lacking. Moreover, despite obvious advancements, the specificity of most biomarkers remains within the 80–90% range with even lower sensitivity, creating the need for more advanced learning models where multiple data points can be combined to increase the overall diagnostic yield. Despite studies highlighting that serum biomarkers can differentiate lung cancer from healthy subjects, more data on patient-centered outcomes, healthcare costs, and morbidity/mortality benefits are needed to encourage routine incorporation into clinical practice. Perhaps data on mortality, missed cancer rates, and/or downstream harms would provide sufficient evidence for incorporation into guidelines. Accessibility, cost, and clinical practice inertia are additional factors that impede widespread utilization in clinical practice. Randomized clinical trials (RCT) are needed to generate high quality clinical utility data.

### 4.5. Coming Soon: Altitude and Nightingale Trials

The ALTITUDE trial will evaluate whether a blood-based integrated classifier (Nodify XL2) can significantly reduce invasive procedures in patients with IPNs while ensuring no increase in the proportion of malignant nodules referred for CT surveillance (Table 4). The study is a multicenter randomized trial that includes more than 2000 patients > 40 years of age with incidentally identified pulmonary nodules. Patients will be randomized 2:1 to receive Nodify XL2 results, whereas the control arm will follow standard of care treatment. Patients will be followed for two years [32].

The NIGHTINGALE trial is also a multicenter RCT that will evaluate the use of the Percepta Nasal Swab to determine the risk of lung cancer when a lung nodule is detected either incidentally or via LDCT (Table 4). This study will include patients aged 29–85 years with a new lung nodule ≤ 30 mm and who have consumed >100 cigarettes in their lifetime [33].

### 4.6. Utilization of Biomarkers at Our Center

At our center, biomarkers are part of the standard management algorithm for both incidentally detected and screen-detected IPNs. Biomarkers are used strictly within validation criteria and incorporated into the shared decision-making discussion along with other diagnostic testing, such as CT and PET imaging. They are used in conjunction with these other tests, not instead of them. Our general workflow is to determine a plan of care, either biopsy or surveillance, at the time of the consultation in the clinic. Biomarker testing is then obtained. Based on the results, the plan of care may change. For example, if a biopsy was planned but biomarker testing suggests the nodule is low risk, then the biopsy will be cancelled, and short-term surveillance imaging will be pursued instead. Even if the plan does not change, confidence in the original plan of care may increase for both the patient and the provider.

## 5. Conclusions

Triage of IPNs has not evolved substantially in several decades. Although numerous risk calculators have been developed, none has consistently proven superior to clinical judgment. Clinicians have naturally erred on the side of caution, leading to many benign nodules being biopsied, causing unnecessary health care expenditure and risk. Various biomarkers have been developed to risk-stratify IPNs. While most have demonstrated good diagnostic accuracy, there are limited, relatively low-quality data demonstrating clinical utility. The first multicenter prospective randomized biomarker RCT (ALTITUDE) designed to assess clinical utility is underway now. As more high-quality data emerge, guidelines will need to address how and where biomarkers should be incorporated into the IPN management algorithm. Currently, biomarkers work best as a complementary tool; they do not obviate the need for diagnostic imaging or other adjudication strategies. They simply serve as an additional data point to help clinicians make the most responsible management decisions for their patients. Although we live in an era of robotic bronchoscopy and cone beam CT imaging, it is imperative that clinicians remember that just because you can do something does not necessarily mean you should.

## Figures and Tables

**Table 1:** Overview of commercially available options.

Name	Assay/Methods	Assay Measurement/Variables Measured	Biomarker Source	Year Introduced	Company
Nodify XL2	MRM-MS	Two plasma proteins + clinical factors	Blood	2019 (precursors in 2013 & 2017)	Biodesix (Louisville, CO, USA)
Nodify CDT	ELISA	Seven autoantibodies	Blood	2020 (precursor 2011)	Biodesix
LungLifeAI	AI + FISH	Circulating genetically abnormal cells	Blood	2024 (precursor 2023)	Circulogene (Birmingham, AL, USA)
CyPath Lung	Flow cytometry	Tumor & tumor-associated cells + age	Sputum	2023	bioAffinity (San Antonio, TX, USA)
Lung Cancer Predictor (LCP)	Convolutional neural network	Radiomic signature	CT scan	2021	Optellum (Oxford, UK)

**Table 2:** Clinical factors pertaining to commercially available options.

Name	Eligibility Criteria	Turnaround Time	Utilization Point	Results
Nodify XL2	Age > 40 8–30 mm <50% risk of malignancy No h/o lung cancer No h/o non-lung cancer within the past 5 years	5 days	Pre-biopsy	Percentage
Nodify CDT	Age > 40 8–30 mm <65% risk of malignancy No h/o cancer	1 day	Pre-biopsy	Percentage
LungLifeAI	Age < 80 <15 mm Upper lobe location	5–7 days	Pre-biopsy	Binary (increased or decreased)
CyPath Lung	Age > 50 6–20 mm Current or former smokers	3 days	Pre-biopsy	Percentage
Lung Cancer Predictor (LCP)	Age > 35 5–30 mm No h/o lung cancer within the past 5 years	<10 s	Pre-biopsy	Risk score 1–10

**Table 3:** How commercially available biomarkers are used. Also summarizes outcomes of clinical accuracy and utility studies. sens = sensitivity; spec = specificity; NPV = negative predictive value; PPV = positive predictive value.

Name	How Test Is Used	Clinical Accuracy Studies	Clinical Utility Studies
Nodify XL2	Rule-out (NPV 98%)	Yes (97% sens)	Yes (ORACLE 2023; CLARIFY & ALTITUDE ongoing)
Nodify CDT	Rule-in (PPV 78%)	Yes (98% spec)	No
LungLifeAI	Rule-in (PPV 80%)	Yes (77% sens, 74% spec)	No
CyPath Lung	Rule-out (NPV 99%)	Yes (92% sens, 87% spec)	No (trial in startup)
Lung Cancer Predictor (LCP)	N/A	Yes	Yes (single center and/or retrospective)

**Table 4:** Biomarkers currently in clinical trials. PN = pulmonary nodule.

Name	Company	Trial Name	Details	Preliminary Results
Percepta Nasal Swab	Veracyte (South San Francisco, CA, USA)	NIGHTINGALE	2400 patients (completed August 2025) >90 academic & community sites	58% of malignant PNs were labeled high risk vs. 37% for physician-assessed risk 42% of malignant PNs were labeled moderate risk No malignant PNs were labeled as low risk
Nodify XL2	Biodesix (Boulder, CO, USA)	ALTITUDE	Estimated enrollment 2000 patients 25 participating sites	None

**Table 5:** Commercial biomarkers that have been taken off the market.

Name	Company	Reason	Discontinuation Date
Reveal & Knodule ID	MagArray (Milpitas, CA, USA)	Operations suspended because of financial insolvency	Pre-2024
Percepta GSC	Veracyte	Resources re-prioritized around Percepta Nasal Swab	2024

## Data Availability

No new data were created or analyzed in this study. Data sharing is not applicable to this article.
